# Integration of 2D/3D deep learning and radiomics for predicting lymphovascular invasion in T1-stage invasive lung adenocarcinoma: a multicenter study

**DOI:** 10.3389/fonc.2025.1631013

**Published:** 2025-10-02

**Authors:** Xiuhua Peng, Shan Pi, Hongxing Zhao, Hupo Bian, Wenhui Li, Dongping Deng, Wenjian Xing, Haihua Hu, Shiyu Zhang, Pengliang Xu, Hanfeng Pan

**Affiliations:** ^1^ Department of Radiology, The First People’s Hospital of Huzhou, Huzhou, China; ^2^ Department of Radiology, The Third Affiliated Hospital, Sun Yat-Sen University, Guangzhou, China; ^3^ Huzhou Key Laboratory of Precise Diagnosis and Treatment of Urinary Tumors, Huzhou, China; ^4^ Department of Thoracic Surgery, The First People’s Hospital of Huzhou, Huzhou, China; ^5^ Department of Radiology, Linghu Hospital, Second Medical Group of Nanxun District, Huzhou, China; ^6^ Department of Radiology, Zhebei Mingzhou Hospital of Huzhou, Huzhou, China; ^7^ Department of Radiology, Xishan People’s Hospital of Wuxi, Wuxi, China

**Keywords:** invasive lung adenocarcinoma, deep learning, radiomics, lymphovascular invasion, artificial intelligence

## Abstract

**Introduction:**

Accurate prediction of the lymphovascular invasion (LVI) status in patients with T1-stage invasive lung adenocarcinoma (LUAD) is crucial for treatment decision-making. Currently, there is a lack of highly efficient and precise prediction models.

**Methods:**

In this retrospective study, 334 patients with T1-stage invasive LUAD who underwent radical surgery from four academic medical centers were included. Conventional radiomic features, two-dimensional deep learning (2D DL) features, and three-dimensional deep learning (3D DL) features were extracted from the tumor regions of the patients’ CT images. Corresponding prediction models were constructed, and these features were integrated to develop a combined model for identifying the LVI status. The performance of the model was evaluated by calculating the area under the receiver operating characteristic (ROC) curve (AUC), and the net benefit of the models was compared using decision curve analysis (DCA).

**Results:**

The combined model demonstrated excellent performance in distinguishing the LVI status, with its predictive ability superior to that of individual models. The AUC values for the training set, internal validation set, and external test set reached 0.958 (95% CI: 0.9294 - 0.9863), 0.886 (95% CI: 0.7938 - 0.9786), and 0.884 (95% CI: 0.8277 - 0.9401), respectively. DCA showed that the net benefit provided by the combined model was higher than that of other radiomic models.

**Conclusions:**

The combined model integrating radiomics, 2D DL, and 3D DL exhibits excellent performance in predicting the LVI status of patients with T1-stage invasive LUAD, and can provide key information for clinical treatment decision-making.

## Introduction

Lung cancer is a leading cause of cancer-related deaths worldwide (1). Non-small cell lung cancer (NSCLC) accounts for 85% to 90% of all lung cancers, with lung adenocarcinoma (LUAD) being the most common histological subtype within NSCLC (2). Lymphovascular invasion (LVI) encompasses both microvascular invasion (MVI) and lymphatic vessel invasion, referring to the invasion of microvessels and/or lymphatic vessel walls or the presence of tumor cell clusters within their lumens, which can only be observed microscopically (3). The presence of LVI in malignant tumors indicates that cancer cells have migrated, marking a critical step in tumor metastasis. LVI has been established as a poor prognostic factor in various malignancies and is an independent indication for postoperative chemotherapy and radiotherapy. For lung cancer patients classified as early-stage or pathological stage with positive LVI, lobectomy offers better clinical outcomes. It reduces the risk of postoperative tumor recurrence and metastasis compared to sublobar resection (4, 5). Due to the difficulty in obtaining tumor stroma-containing microvessels or lymphatic vessels through needle biopsy, LVI information is generally not obtainable solely from such biopsies. Therefore, preoperative assessment of LVI status in LUAD remains challenging, and pathological diagnosis of postoperative specimens is currently the only method to determine LVI status (6, 7). Due to the delays associated with pathological diagnosis, accurate preoperative evaluation of LVI in T1-stage LUAD is crucial for clinical decision-making and individualized treatment for T1-stage lung cancer patients, making it a focal point of current research both domestically and internationally.

Some researchers suggest that specific preoperative computed tomography(CT) findings, such as nodule composition, consolidation to tumor ratio (C/T ratio), spiculated margins, abnormal veins, peritumoral stromal thickening, and pleural contact, are associated with the occurrence of LVI (8, 9). Choe et al. (8) also noted that LVI occurs only in solid nodules or part-solid nodules with solid components more significant than 10 mm, with peritumoral stromal thickening and pleural contact identified as independent predictors of LVI. However, the evaluation of imaging features is heavily influenced by the experience of radiologists and their understanding of different findings, leading to significant subjective reliance and poor reproducibility. These factors limit the effectiveness of traditional imaging in preoperatively predicting LVI in lung cancer.

Radiomics, as a robust imaging biomarker, can non-invasively assess tumor heterogeneity that is not detectable by the human eye and can reflect intratumoral angiogenesis (10). Several studies have applied radiomic and related combined models to predict LVI status in NSCLC, achieving promising results (11–13). With the rapid development of deep learning (DL), DL features have complemented traditional radiomic features in medical imaging (14). DL imaging features extracted based on convolutional neural networks (CNN) are used to construct feature signatures and have been shown to enhance model performance in various clinical tasks (15). DL has been widely applied in NSCLC research, including lung nodule classification, lung cancer metastasis prediction, gene mutation prediction, airspace dissemination prediction, and treatment efficacy assessment (16–20).

Traditional radiomics analyzes tumor texture features by considering the entire tumor as the region of interest (ROI). In contrast, feature extraction in DL is a critical step within DL models. When selecting the ROI, we face a trade-off. Tumors appear across multiple slices in CT images, allowing for the extraction of features from a slice representing the maximum cross-sectional diameter of the tumor (two-dimensional,2D) or from a cube encompassing the entire tumor volume (three-dimensional,3D). Compared to 3D ROIs, 2D ROIs are more accessible to obtain, require less time and labor, are less complex, and have faster computational speeds. Intuitively, 3D DL features may provide more comprehensive information about the entire tumor. Previous studies have employed 2D and 3D ROIs, but their performance differences have yielded inconsistent results. It remains unclear whether the time-consuming and labor-intensive 3D DL analysis is inherently more valuable than 2D DL analysis, and it is uncertain whether DL features necessarily outperform texture features. In conclusion, currently, there is no reported study to prove which imaging method, traditional radiomics, 2D DL, or 3D DL, is more accurate in predicting LVI in T1-stage LUAD.

This study assesses the correlation between chest CT imaging features and LVI status in T1-stage invasive LUAD. We will conduct a DL radiomics study based on chest CT images, constructing traditional radiomic, 2D DL, 3D DL, and combined models. We will compare the diagnostic performance of these different models to provide the best predictive model for LVI status in T1-stage invasive LUAD.

## Materials and methods

### Study design

**Figure 1:**
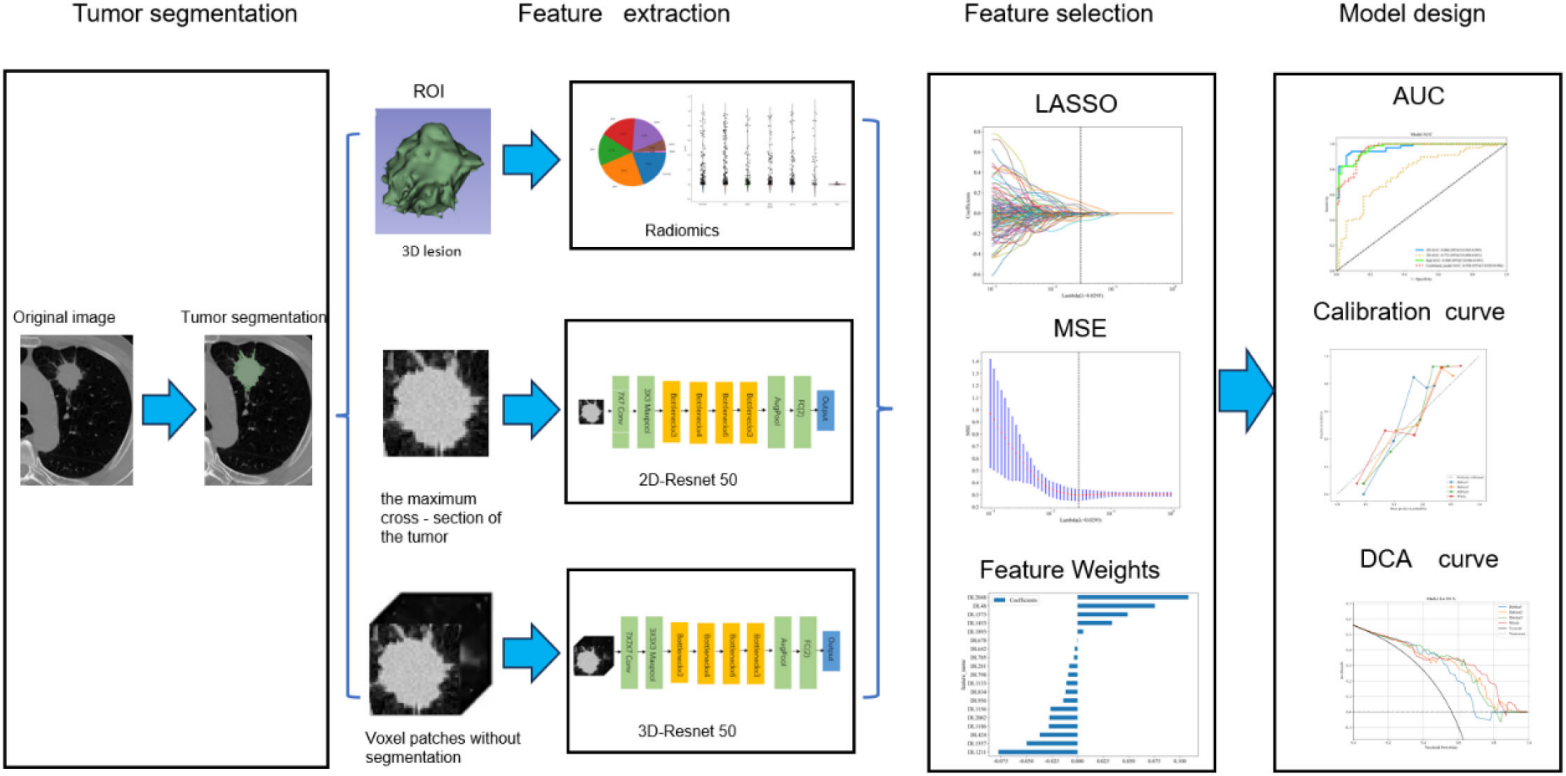
Workflow of radiomics analysis.

Our study introduced four radiomic models: a traditional radiomic model, a 2D DL model, a 3D DL model, and a combined model of the three. The radiomic analysis was conducted through several steps: image segmentation, feature extraction, feature selection, feature construction, and validation (**Figure 1**).

### Patient characteristics

**Figure 2:**
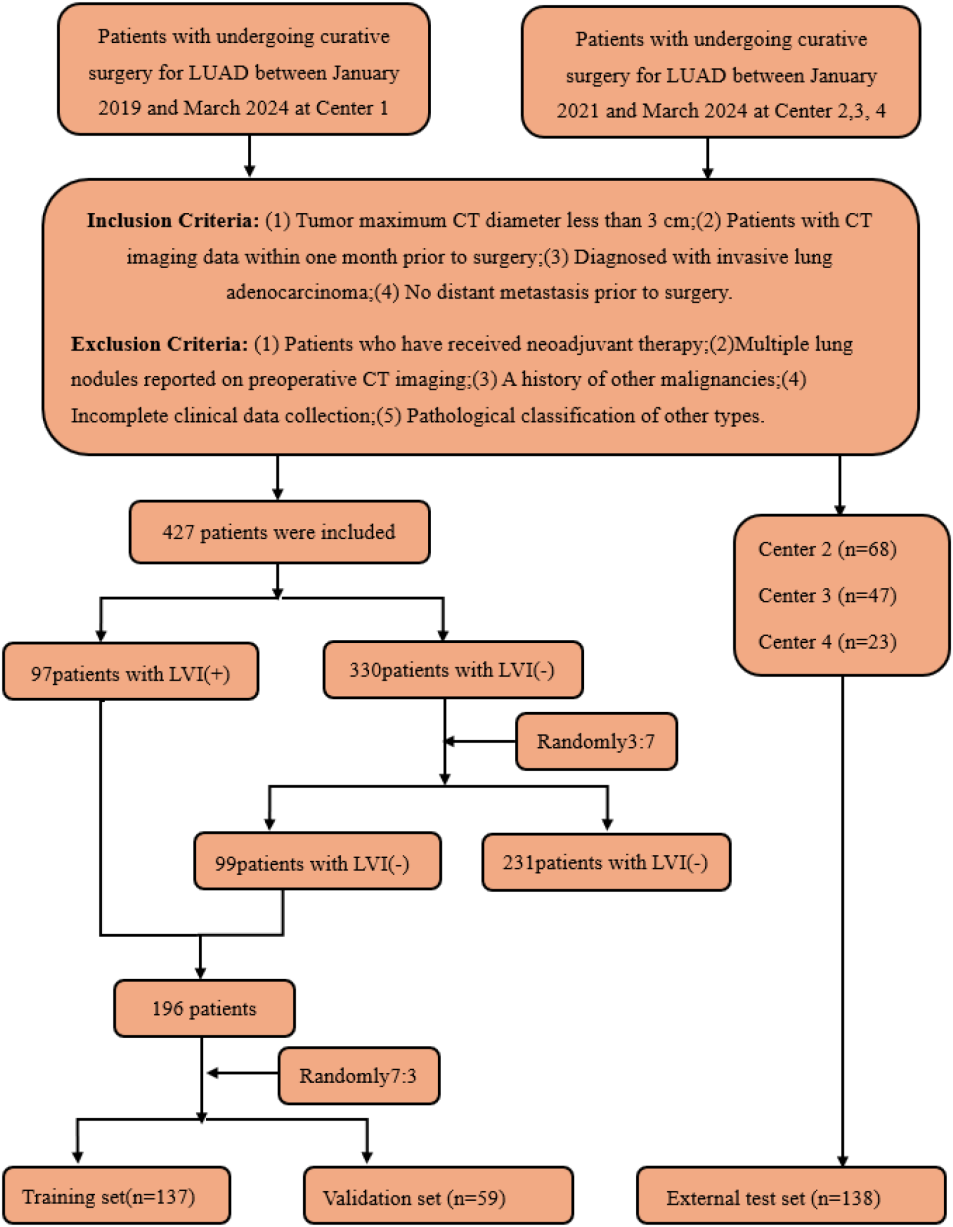
Flow diagram of the enrolment of patients. LVI, lymphovascular invasion; LVI (+), positive for LVI; LVI (-), negative for LVI.

This retrospective study included T1-stage invasive LUAD patients who underwent radical surgery at four academic medical centers. Preoperative CT images and clinical pathological data were collected. Inclusion criteria were: (1) maximum tumor diameter on CT less than 3 cm; (2) CT imaging data obtained within one month before surgery; (3) diagnosis of invasive LUAD; (4) no distant metastasis before surgery. Exclusion criteria included: (1) patients who received neoadjuvant therapy; (2) patients with multiple pulmonary nodules reported on preoperative CT; (3) patients with a history of other malignant tumors; (4) patients with incomplete clinical data; (5) patients with pathological types classified as other types. A total of 334 patients were included in this study (**Figure 2**). In this study, 334 patients with T1-stage invasive LUAD from four academic medical centers were enrolled. All these patients underwent radical surgery and had preoperative CT images as well as clinicopathological data available. In Center 1, there were 427 eligible patients, among whom only 97 patients had LVI positivity, while as many as 330 patients had LVI negativity. There was a significant imbalance in the sample sizes.

This imbalance might lead to insufficient learning of the minority category during the model training process, which in turn could affect the performance and generalization ability of the model. For instance, the recognition accuracy of the minority category in prediction was relatively low.

To address this issue, this study referred to the validated sample allocation strategy (21). A total of 99 samples were randomly selected from the negative group in Center 1 at a ratio of 3:7, so as to make the ratio of LVI-positive to LVI-negative patients in the training set approach 1:1. The reason for adopting the random sampling method at a ratio of 3:7 for the negative group to select cases was that it enabled us to exactly sample 99 cases, and this number was close to that of the LVI-positive group. This effectively balanced the proportion of the two types of samples in the training set and avoided the learning bias caused by sample imbalance during the model training process.

Meanwhile, a strict random selection method was employed to exclude, to the greatest extent possible, the subjective biases that might be introduced by human selection, ensuring that the selected data could truly and objectively reflect the overall characteristics, thus enhancing the representativeness and universality of the data.

Eventually, these patients were allocated reasonably: 137 patients (68 positive and 69 negative) were included in the training set, 59 patients (29 positive and 30 negative) served as the internal validation set, and 138 patients (48 positive and 90 negative) from Centers 2, 3, and 4 constituted the external test set. Through such a sample distribution, not only was a reasonable sample size ensured for each dataset, but also the generalization ability of the model was effectively improved with the aid of multi-center external validation.

### Histopathological evaluation

Each case’s pathological specimen was independently reviewed by two experienced thoracic pathologists (with 5 and 10 years of diagnostic experience, respectively), blinded to the clinical information. In cases of disagreement regarding the findings, consensus was reached through discussion. As observed microscopically, LVI was defined as the invasion of microvessels and/or lymphatic vessel walls or tumor cell clusters within their lumens.

Pathological data were collected for each patient, including clinical pathological staging (according to the 8th AJCC TNM staging system), histopathological grading (using the 2015 IASLC/ATS/ERS LUAD classification, which categorizes tumors into lepidic predominant adenocarcinoma (LPA), acinar predominant adenocarcinoma (APA), papillary predominant adenocarcinoma (PPA), micropapillary predominant adenocarcinoma (MPA), solid predominant adenocarcinoma (SPA), and other rare patterns), invasion of visceral pleura, STAS, lymph node metastasis, and other relevant factors.

### Clinicopathological variables

Clinical pathological data were collected for each patient, including clinical information such as age, sex, Carcinoembryonic Antigen (CEA), Cancer Antigen 125(CA125), tumor location, surgical method, and the presence of emphysema. Pathological data included pleural invasion, pathological classification, grading, Ki-67, STAS, lymph node metastasis, and other relevant factors.

### CT acquisition and interpretation

The chest scan was performed with a German Siemens Definition AS 64-row 128-slice spiral CT. Scan from the thoracic entrance to the diaphragm level. The subjects were placed in the supine position and held their breath after deep inhalation. Scanning parameters: tube voltage 120kV, tube current 120mA, window width 1300-1500, window position: -600~-700, pitch 1.0, frame rotation time 0.33S/360 degrees. Lung window reconstruction was performed using the lung method with a reconstruction thickness of 1.25mm and layer spacing of 1.25mm. Mediastinal window reconstruction thickness and layer spacing were 5mm.

Two experienced thoracic radiologists (with 5 and 10 years of experience in lung nodule diagnosis, respectively) assessed traditional imaging features. They independently reviewed the CT images while blinded to the pathological and clinical information. In cases of disagreement regarding the findings, consensus was reached through discussion. The evaluated imaging features included composition (solid, part-solid, or ground-glass), maximum tumor diameter, lobulation, spiculation, vacuole sign, air bronchogram sign, vascular clustering, pleural retraction, and peritumoral ground-glass opacification.

### Conventional radiomics ROI segmentation and feature extraction

Since different CT scanners were used in this study, it is necessary to preprocess the images before performing segmentation and feature extraction to make the radiomics features more robust and more suitable for further analysis. First, in the image preprocessing step, all images were resampled to a voxel size of 1 mm × 1 mm × 1 mm to standardize the voxel spacing. Subsequently, Z-score normalization (zero-mean normalization) was employed to standardize the data. Two experienced radiologists independently performed image segmentation without knowing the patients’ pathological conditions. Radiologist A (with five years of experience) manually delineated the ROIs layer by layer using the open-source software ITK-SNAP (version 3.8.0, http://www.itksnap.org). Radiologist B (with ten years of experience) reviewed all ROIs manually segmented by Radiologist A and manually removed tumor regions overlapping with soft tissue, bone, and mediastinum in the chest wall. If there is a disagreement between Doctor A and Doctor B during the segmentation process, Doctor C, with rich professional experience, will be introduced for intervention. When re-segmenting the controversial area, Doctor C will comprehensively consider various factors such as the morphology and location of the tumor, as well as the imaging features at different levels, so as to ensure the accuracy of the segmentation result.

Traditional radiomic features were extracted using PyRadiomics, a Onekey AI software suite component. These features were categorized into three types: geometric features, intensity features, and texture features. Geometric features were used to describe the 3D shape characteristics of the tumor; intensity features described the first-order statistical distribution of voxel intensities within the tumor; and texture features reflected the patterns of intensity or second- and higher-order spatial distributions. The extraction of texture features utilized various methods, including gray level co-occurrence matrix (GLCM), gray level run length matrix (GLRLM), gray level size zone matrix (GLSZM), gray level dependence matrix (GLDM), and neighborhood gray-tone difference matrix (NGTDM).

### DL ROI segmentation and feature extraction

Since different CT scanners were used in this study, before performing tumor annotation and DL analysis, it is necessary to standardize the relevant processes and process the images to reduce the differences caused by different devices and improve the accuracy and reliability of the research results. To achieve this goal, the following key steps were taken:

(1) In the tumor annotation stage, the reconstructed CT images were imported into the ITK-SNAP software (Version 3.8.0, http://www.itksnap.org). Two radiologists with 5 years of experience independently carried out the annotation under the lung window setting (average value: -450 Hounsfield Unit (HU); width: 1500 HU). For the inconsistent situations among observers during the annotation process, a senior radiologist coordinated and solved them to ensure the consistency of the annotation. In terms of the selection of ROI, different strategies were adopted according to different types of neural networks. The 2D CNN selected the slice with the largest tumor area, while the 3D CNN used the bounding box containing the entire tumor volume for annotation.
(2) In terms of image preprocessing, to eliminate the differences in voxel length in the images, all images were interpolated to unify the voxel spacing to (1 mm × 1 mm × 1 mm) before being input into the network. At the same time, the tumor images were standardized to HU values with the help of the DICOM header information, and a threshold was set to prevent extreme values from affecting the analysis results. In addition, the mean and variance of the 3D tumor images calculated in the training set were standardized through the Z-score method, thus promoting the learning of the network and enhancing the adaptability of the model to the images obtained from different CT scanners. Furthermore, this study utilized the ResNet50 deep convolutional network architecture (3D version) for feature extraction, effectively addressing the degradation problem in deep networks through residual blocks.

### Feature selection and model construction

Traditional radiomic feature sets, 2D DL feature sets, and 3D DL feature sets were obtained through the feature extraction methods above. All patients were randomly stratified by center into various cohorts (**Figure 2**). Huzhou First People’s Hospital patients were divided into training and internal validation sets at a 7:3 ratio. Additionally, all patients from Huzhou Mingzhou Hospital, Huzhou Nanxun District Second Medical Group Linghu Hospital, and Wuxi Xishan People’s Hospital were considered the external test set. Before feature selection, the features in the training set were normalized to scale different features to the same magnitude. Subsequently, feature selection was performed in three steps: first, all radiomic features underwent Mann-Whitney U tests for feature selection, retaining only those with a p-value less than 0.05. Subsequently, for highly redundant features, the Spearman rank correlation coefficient was calculated to assess the correlation between features; if the coefficient between any two features exceeded 0.9, one feature was retained. Finally, the Least Absolute Shrinkage and Selection Operator (LASSO) regression model(a statistical method for selecting key features by shrinking feature coefficients) was utilized to construct feature signatures on the exploratory dataset. By adjusting the regularization weight λ, LASSO shrinks all regression coefficients towards zero, setting many irrelevant feature coefficients to precisely zero. To identify the optimal λ, a minimum standard 10-fold cross-validation was employed, with the final value of λ resulting in the smallest cross-validation error. Features with non-zero coefficients were retained for regression model fitting and combined into a radiomic signature. Subsequently, we calculated the patients’ radiomic scores (rad scores, RS) by linearly combining the retained features, weighted by their model coefficients. After feature selection, traditional radiomic, 2D DL, 3D DL, and combined feature sets were constructed.

Using Onekey AI software, Multi-Layer Perceptron (MLP) models were constructed on the training set feature sets for traditional radiomic, 2D DL, 3D DL, and combined models, tested on internal and external validation sets. The architecture of the MLP classifier includes an input layer, hidden layers, and an output layer. In this study, the input layer receives multi-dimensional input data from traditional radiomics features, 2D DL features, and 3D DL features. The hidden layers are composed of multiple fully connected layers. Each fully connected layer is followed by a Rectified Linear Unit (ReLU) activation function(a computational method that enhances the model’s ability to learn non-linear features), which is used to extract non-linear feature representations. The output layer, through a fully connected layer and a Sigmoid activation function, maps the final features to the probability value of LVI being positive, with the value ranging from 0 to 1. The model uses the binary cross-entropy loss function to evaluate the prediction error and updates the parameters through the Adam optimizer to minimize the loss function. Through this hierarchical structure and non-linear transformation, the MLP can effectively learn complex feature relationships and is suitable for binary classification tasks. The MLP model was consistently used throughout this study to ensure comparability. MLPs are advantageous for learning nonlinear relationships, suitable for multitasking applications, structurally simple, easily adjustable, and capable of automatic feature extraction. This model has demonstrated outstanding performance in many practical applications due to its efficiency and robustness.

### Statistical methods

Statistical analyses were conducted using Onekey AI software and R software version 4.0.2. Univariate and multivariate logistic regression analyses were performed to compare clinical CT and pathological features between LVI-positive and LVI-negative patients, identifying independent predictors of LVI positivity. Receiver operating characteristic (ROC) curves were plotted, and the area under the curve (AUC), 95% confidence interval (CI), accuracy (ACC), specificity (SPE), sensitivity (SEN), positive predictive value (PPV), and negative predictive value (NPV) were calculated. The performance of each model was evaluated, and DeLong’s test was used to compare their differences. Calibration curves were plotted to assess the model’s calibration. Decision curve analysis (DCA) was employed to evaluate the clinical value of the models. A p-value of <0.05 was considered statistically significant.

### Ethical statement

This study was conducted by the Declaration of Helsinki and received approval from the Ethics Committee of Huzhou First People’s Hospital. Additionally, due to its retrospective nature, the Institutional Review Board exempted the requirement for prior informed consent from all participants.

## Results

### Baseline characteristics of the patients

This study included a total of 334 patients with clinical T1 stage invasive lung adenocarcinoma, comprising 137 patients in the training set (68 LVI-positive and 69 LVI-negative), 59 patients in the internal validation set (29 LVI-positive and 30 LVI-negative), and 138 patients in the external testing set (48 LVI-positive and 90 LVI-negative). Patient clinical data, CT characteristics, and pathological information were recorded. The clinical baseline characteristics of all patients are presented in **Table 1**.

**Table 1:** Baseline characteristics of patients in the training cohort and test cohort.

Variable	Training set (n = 137)		P	Validation set (n = 59)		P	External test set (n = 138)		P
	LVI(-)	LVI (+)		LVI(-)	LVI (+)		LVI(-)	LVI (+)	
Age	64.99 ± 10.21	63.79 ± 8.70	0.262	63.33 ± 9.55	66.90 ± 8.80	0.142	63.57 ± 11.71	61.34 ± 11.25	0.134
Maximum tumor diameter(mm)	1.46 ± 0.57	2.11 ± 0.76	<0.001	1.47 ± 0.65	1.97 ± 0.63	<0.001	1.34 ± 0.52	2.06 ± 0.58	<0.001
Gender			0.201			0.104			0.274
Male	27(39.13)	35(51.47)		14(46.67)	20(68.97)		38(41.76)	25(53.19)	
Female	42(60.87)	33(48.53)		16(53.33)	9(31.03)		53(58.24)	22(46.81)	
Lymph node metastasis			<0.001			0.976			<0.001
No	68(98.55)	53(77.94)		29(96.67)	27(93.10)		91(100.00)	32(68.09)	
Yes	1(1.45)	15(22.06)		1(3.33)	2(6.90)		0	15(31.91)	
Emphysema			0.055			0.945			0.012
No	54(78.26)	42(61.76)		24(80.00)	22(75.86)		89(97.80)	40(85.11)	
Yes	15(21.74)	26(38.24)		6(20)	7(24.14)		2(2.20)	7(14.89)	
Location			0.345			0.452			0.125
RUL	26(37.68)	19(27.94)		10(33.33)	6(20.69)		23(25.27)	10(21.28)	
RML	9(13.04)	5(7.35)		5(16.67)	6(20.69)		5(5.49)	5(10.64)	
RLL	7(10.14)	8(11.76)		5(16.67)	2(6.09)		24(26.37)	10(21.28)	
LUL	14(20.29)	23(33.82)		7(23.33)	12(41.38)		32(35.16)	12(25.53)	
LLL	13(18.84)	13(19.12)		3(10.00)	3(10.34)		7(7.69)	10(21.28)	
Surgical approach			<0.001			0.012			<0.001
Lobectomy	48(69.57)	18(26.47)		18(60.00)	7(24.14)		70(76.92)	12(25.53)	
Segmentectomy	21(30.43)	50(73.53)		12(40.00)	22(75.86)		21(23.08)	35(74.47)	
Differentiation grade			<0.001			0.003			<0.001
High-grade	17(24.64)	0		3(10.00)	0		15(16.48)	1(2.13)	
Intermediate-grade	47(68.12)	46(67.65)		25(83.33)	17(58.62)		73(80.22)	29(61.70)	
Low-grade	5(7.25)	22(32.35)		2(6.67)	12(41.38)		3(3.30)	17(36.17)	
Histological type			<0.001			0.116			<0.001
APA	45(65.22)	32(47.06)		19(63.33)	11(37.93)		75(82.42)	28(59.57)	
PPA	0	11(16.18)		1(3.33)	4(13.79)		1(1.10)	9(19.15)	
MPA	5(7.25)	4(5.88)		1(3.33)	5(17.24)		4(4.40)	3(6.38)	
SPA	10(14.49)	21(30.88)		7(23.33)	9(31.03)		11(12.09)	4(8.51)	
LPA	8(11.59)	0		1(3.33)	0		0	3(6.38)	
STAS			<0.001			<0.001			<0.001
Negative	59(85.51)	27(39.71)		30(100.00)	8(27.59)		89(97.80)	15(31.91)	
Positive	10(14.49)	41(60.29)		0	21(72.41)		2(2.20)	32(68.09)	
KI67			<0.001			<0.001			<0.001
<20%	67(97.10)	48(70.59)		28(93.33)	14(48.28)		86(94.51)	19(40.43)	
≥20%	2(2.90)	20(29.41)		2(6.67)	15(51.72)		5(5.49)	28(59.57)	
Pleural invasion			<0.001			0.162			0.017
Negative	65(94.20)	48(70.59)		27(90.00)	21(72.41)		83(91.21)	35(74.47)	
Positive	4(5.80)	20(29.41)		3(10.00)	8(27.59)		8(8.79)	12(25.53)	
Lobulated sign			<0.001			0.286			<0.001
Negative	33(47.82)	12(17.65)		11(36.67)	6(20.69)		43(47.25)	6(12.77)	
Positive	36(52.17)	56(82.35)		19(63.33)	23(79.31)		48(52.75)	41(87.23)	
Vacuolated sign			0.939			0.710			<0.001
Negative	39(56.52)	37(54.41)		19(63.33)	16(55.17)		45(49.45)	5(10.64)	
Positive	30(43.48)	31(45.59)		11(36.67)	13(44.83)		46(50.55)	42(89.36)	
Peripheral GGO			0.411			0.446			1
Negative	53(76.81)	47(69.12)		19(63.33)	22(75.86)		75(82.42)	38(80.85)	
Positive	16(23.19)	21(30.88)		11(36.67)	7(24.14)		16(17.58)	9(19.15)	
Vascular bundle			0.675			0.905			0.029
Negative	28(40.58)	31(45.59)		13(43.33)	14(48.28)		65(71.43)	24(51.06)	
Positive	41(59.42)	37(54.41)		17(56.67)	15(51.72)		26(28.57)	23(48.94)	
Spiculation			0.146			0.501			0.010
Negative	44(63.77)	34(50.00)		19(63.33)	10(34.48)		80(87.91)	32(68.09)	
Positive	25(36.23)	34(50.00)		11(36.67)	19(65.52)		11(12.09)	15(31.91)	
Bronchus sign			0.809			1			0.007
Negative	49(71.01)	46(67.65)		22(73.33)	21(72.41)		75(82.42)	28(59.57)	
Positive	20(28.99)	22(32.35)		8(26.67)	8(27.59)		16(17.58)	19(40.43)	
Pleural Indentation			0.009			0.701			0.003
Negative	54(78.26)	38(55.88)		17(56.67)	14(48.28)		60(65.93)	18(38.30)	
Positive	15(21.74)	30(44.12)		13(43.33)	15(51.72)		31(34.07)	29(61.70)	
Nodule type			<0.001			<0.001			<0.001
Solid	8(11.59)	31(45.59)		2(6.67)	14(48.28)		11(12.09)	30(63.83)	
Part solid	45(65.22)	37(54.41)		21(70.00)	14(48.28)		63(69.23)	17(36.17)	
pGGN	16(23.19)	0		7(23.33)	1(3.45)		17(18.68)	0	
CEA			<0.001			0.203			<0.001
Negative	64(92.75)	44(64.71)		25(83.33)	19(65.52)		81(89.01)	29(61.70)	
Positive	5(7.25)	24(35.29)		5(16.67)	10(34.48)		10(10.99)	18(38.30)	
CA125			1			0.986			1
Negative	65(94.20)	65(95.59)		30(100.00)	28(96.55)		87(95.60)	45(95.74)	
Positive	4(5.80)	3(4.41)		0	1(3.45)		4(4.40)	2(4.26)	

Data were presented as mean ± SD, or n (%) unless otherwise stated. L, left lower lobe; LUL, left upper lobe; RLL, right lower lobe; RML, right middle lobe; RUL, right upper lobe; pGGN, pure ground-glass nodule; APA, acinar predominant adenocarcinoma; PPA, Papillary predominant adenocarcinoma; MPA, Micropapillary predominant adenocarcinoma; SPA, solid predominant adenocarcinoma; LPA, lepidic predominant adenocarcinoma; mucinous.

### Clinicopathological and CT features by LVI status

Univariate and multivariate analyses were performed on the clinical characteristics of the training set, and the odds ratios (OR), along with their corresponding p - values, were calculated for each feature (**Table 2**). In the multivariate analysis: For pathological grading, the OR was 0.403, with a 95% confidence interval (CI) of 0.237 - 0.685 and P = 0.005. For STAS, the OR was 2.751, with a 95% CI of 1.223 - 6.190 and P = 0.040. Only pathological grading and STAS were significant (P < 0.05), serving as independent predictors of LVI.

**Table 2:** Univariable and multivariable analysis of clinical features.

Variable	Univariate analysis		Multivariable analysis	
	OR (95%CI)	P	OR (95%)CI	P
Gender	0.786 (0.536-1.151)	0.300		
Age	1.000 (0.995-1.004)	0.848		
Lymph node metastasis	15.001 (2.743-82.023)	0.009	5.797 (0.855-39.330)	0.131
Emphysema	1.733 (1.071-2.954)	0.090		
Location	1.063 (0.947-1.192)	0.384		
Surgical approach	2.381 (1.553-3.651)	0.001	2.119 (1.062-4.229)	0.074
Histological type	0.993 ( 0.862-1.143)	0.932		
Differentiation grade	1.400 (1.101-1.781)	0.021	0.403 (0.237-0.685)	0.005
STAS	4.100 ( 2.296-7.323)	0.000	2.751 (1.223-6.190)	0.040
KI67	10.000 (2.954-33.852)	0.002	4.999 (1.230-20.308)	0.059
Pleural invasion	5.000 (2.032-12.305)	0.003	3.086 (0.991-9.612)	0.103
Maximum tumor diameter	1.184 ( 1.019-1.374)	0.063		
Lobulated sign	1.462 (1.038-2.061)	0.068		
Vacuolated sign	1.033 (0.678-1.575)	0.898		
Peripheral GGO	1.312 ( 0.760-2.266)	0.413		
Vascular bundle sign	0.902 ( 0.621-1.310)	0.651		
Speculation	1.360 (0.882-2.098)	0.243		
Bronchus sign	1.100 (0.662-1.828)	0.758		
Pleural Indentation	2.000 (1.189-3.364)	0.028	1.156 (0.532-2.512)	0.759
Nodule type	0.850 (0.733-0.986)	0.071		
CEA	4.800 (2.138-10.773)	0.001	2.660 (0.937-7.553)	0.123
CA125	0.750 (0.214-2.635)	0.706		

LLL, left lower lobe; LUL, left upper lobe; RLL, right lower lobe; RML, right middle lobe; RUL, right upper lobe; pGGN, pure ground-glass nodule; APA, acinar predominant adenocarcinoma; PPA, Papillary predominant adenocarcinoma; MPA, Micropapillary predominant adenocarcinoma; SPA, solid predominant adenocarcinoma; LPA, lepidic predominant adenocarcinoma.

### Feature selection and radiomics signature development

**Figure 3:**
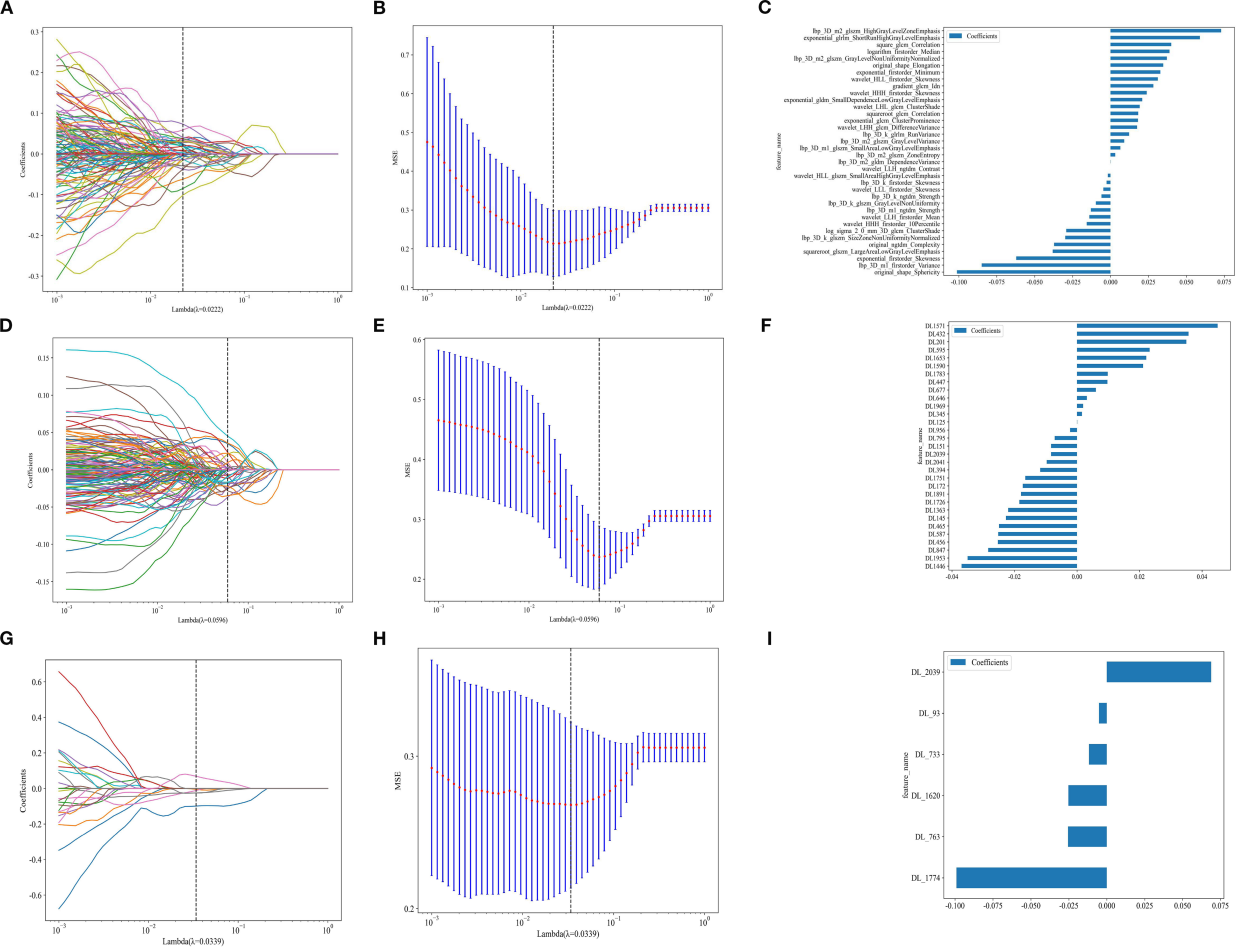
**(A, D, G)** represent the LASSO for radiomics, 2D DL, and 3D DL features. **(B, E, H)**represent the MSE for Rad, 2D DL, and 3D DL features. **(C, F, I)** represent the feature weights for Rad, 2D DL, and 3D DL features.

Radiomic(Rad),2D, and 3D DL features were extracted using CT images. Following the Intraclass Correlation Coefficient (ICC) test results, 1834 radiomic features and 2048 DL features were retained, creating datasets for radiomic features, 2D DL features, and 3D DL features. Each dataset underwent t-tests, Pearson correlation analysis, and LASSO for final selection, resulting in 36, 31, and 6 optimal features, respectively (**Figure 3**). Subsequently, traditional Rad models, 2D DL models, 3D DL models, and a combined model were constructed.

We used an MLP classifier to predict the models for each feature set. Training set: The combined model and the traditional radiomics model showed the best performance (AUC: 0.958, 95%CI: 0.9294-0.9863 and 0.968, 95%CI: 0.9460-0.9909, respectively), followed by the 2D DL model (0.968, 95%CI: 0.9432-0.9933), and the 3D DL model performed the worst (0.772, 95%CI: 0.6939-0.8509).

Internal validation set: The combined model took the lead in performance (AUC = 0.886, 95%CI: 0.7938-0.9786), followed by the traditional radiomics model (0.844, 95%CI: 0.7360-0.9513). The performance of the 2D/3D DL models decreased significantly (0.759, 95%CI: 0.6338-0.8835; 0.740, 95%CI: 0.6131-0.8674).

External test set: The combined model (0.884, 95%CI: 0.8277-0.9401) and the traditional radiomics model (0.870, 95%CI: 0.8084-0.9306) maintained stable performance, while the performance of the 2D DL model decreased significantly (0.613, 95%CI: 0.5186-0.7074), suggesting its weak cross-center generalization ability (**Table 3**).

**Table 3:** Performance of each model of the MLP classifier in predicting LVI.

Model	Dataset	Accuracy	AUC	95% CI	Sensitivity	Specificity	PPV	NPV
Rad	Train	0.891	0.968	0.9460 - 0.9909	0.809	0.971	0.965	0.837
	Validation	0.780	0.844	0.7360 - 0.9513	0.828	0.733	0.750	0.815
	External test	0.810	0.870	0.8084-0.9306	0.809	0.811	0.691	0.890
2D	Train	0.920	0.968	0.9432 - 0.9933	0.926	0.913	0.913	0.926
	Validation	0.712	0.759	0.6338 - 0.8835	0.759	0.667	0.687	0.741
	External test	0.577	0.613	0.5186-0.7074	0.787	0.467	0.435	0.808
3D	Train	0.708	0.772	0.6939 - 0.8509	0.574	0.841	0.780	0.667
	Validation	0.678	0.740	0.6131 - 0.8674	0.828	0.533	0.632	0.762
	External test	0.701	0.691	0.5930 - 0.7895	0.660	0.722	0.554	0.802
Combined	Train	0.891	0.958	0.9294 - 0.9863	0.971	0.812	0.835	0.971
	Validation	0.847	0.886	0.7938 - 0.9786	0.793	0.900	0.885	0.818
	External test	0.810	0.884	0.8277 - 0.9401	0.809	0.811	0.681	0.890

In the training set, the Hosmer-Lemeshow test p-values for all models were greater than 0.05 (2D model: 0.098, 3D model: 0.751, Rad model: 0.292, Combined_model: 0.314), indicating that these models have a high degree of agreement between predicted probabilities and actual outcomes. However, in the internal validation set, the Hosmer-Lemeshow test p-values for the 2D, 3D, and Rad models were also greater than 0.05 (2D model: 0.589, 3D model: 0.870, Rad model: 0.707), suggesting good calibration performance for these models. In contrast, the p-value for the Combined_model was 0.024, which is less than 0.05. In the external test set, all models showed significant prediction bias (P < 0.05), which is consistent with the decreased performance. This confirms the limited generalizability of the models in cross-center scenarios-particularly the combined model, whose calibration bias was already observed in the internal validation phase, may have exacerbated performance fluctuations in the external test set. Future studies will optimize the cross-center robustness of the models through data augmentation and domain adaptation algorithms to improve calibration performance and generalizability.

Through the analysis of DCA curves for the training set, internal validation set, and external test set, we found that the Combined_model provided the highest net benefit across a wide range of threshold probabilities (10% to 50%), indicating its high utility in clinical decision-making. However, the performance of the Combined_model gradually declined as the dataset changed, particularly in the external test set, suggesting limited generalizability. In contrast, the 2D, 3D, and Rad models performed adequately in the training and internal validation sets but showed poorer performance in the external test set, indicating limited clinical applicability. The Treat all strategy performed well at low thresholds but poorly at high thresholds, while the Treat none strategy consistently yielded no net benefit. The combined model has clinical value in same-center scenarios, but its cross-center robustness needs to be prioritized for optimization. Future efforts will focus on two aspects: on one hand, applying a balanced sampling strategy consistent with that of the training set in the preprocessing stage of the external test set to reduce the impact of class imbalance on model generalization; on the other hand, integrating data augmentation and domain adaptation techniques to improve cross-center stability and enhance the reliability of clinical applications.

**Figure 4:**
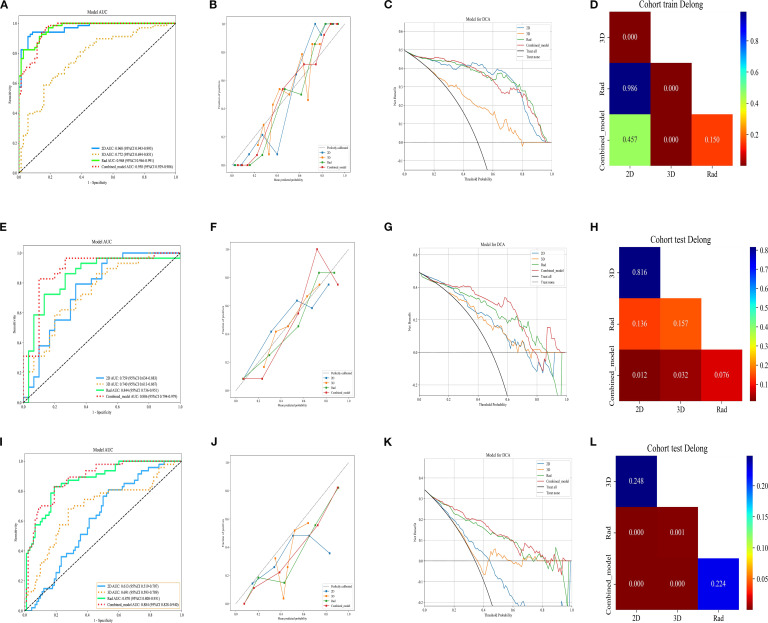
ROC curve of different models in the **(A)** train, **(E)** validation, and **(I)** external test sets, respectively. Calibration curve of different models in the **(B)** train, **(F)** validation, and **(J)** external test sets. DCA curve of different models in the **(C)** train, **(G)** validation, and **(K)** external test sets. Delong test of different models in the **(D)** train, **(H)** validation, and **(l)** external test sets, respectively. Rad, Rad signature; 2D, 2D DL signature; 3D, 3D DL signature; Combined, combined 2D, 3D and Rad signature.

The ROC curves, calibration curves, DCA, and DeLong test for all signatures in the training and testing cohorts are shown in **Figure 4**.

**Figure 5:**
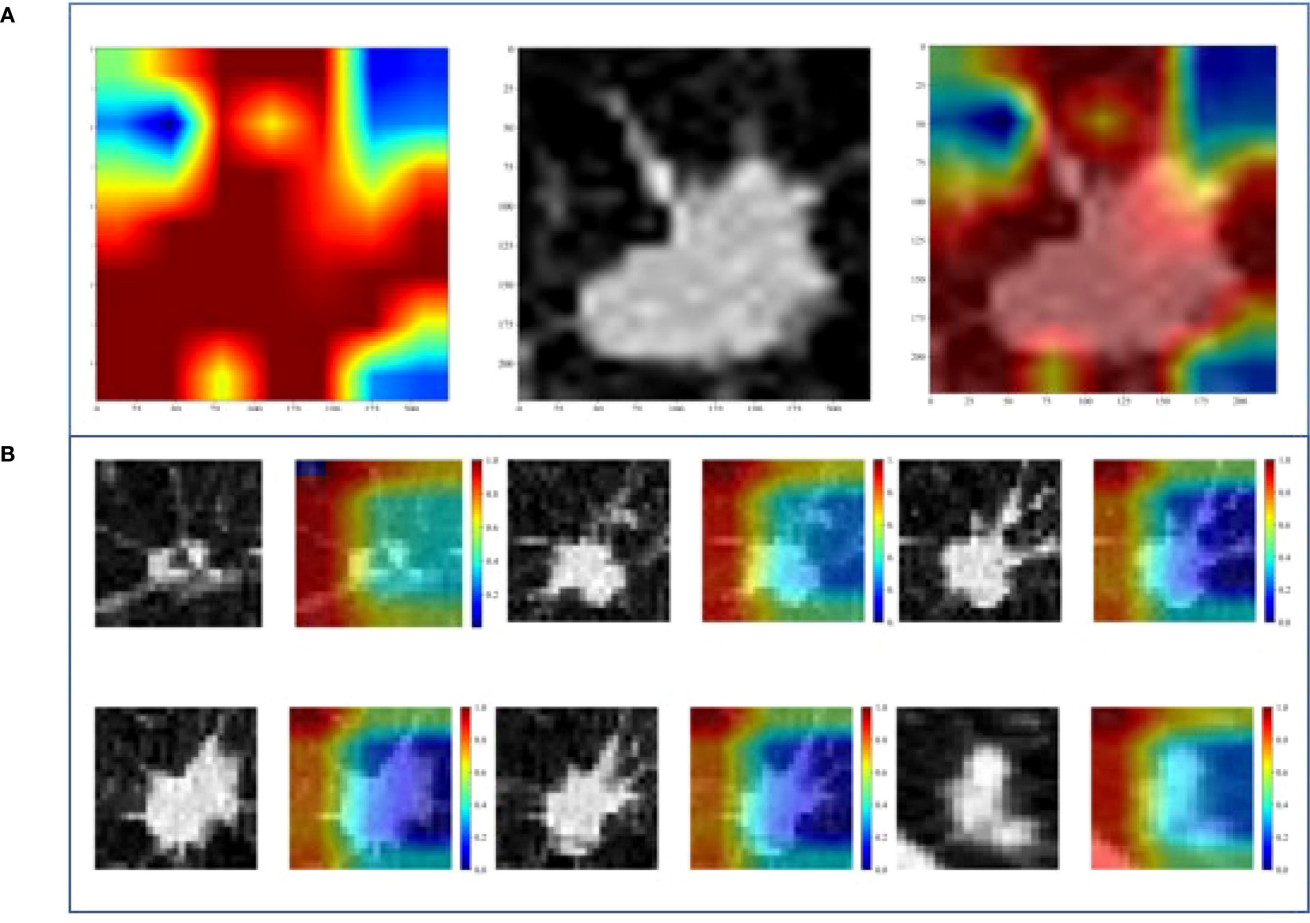
Gradient-weighted class activation mapping (Grad-CAM) of two DL models. **(A)** 2D-DL model, **(B)** 3D-DL model.

**Figure 5** shows the visualization images of two deep learning models.

## Discussion

In this study, we extracted Rad features, 2D DL features, and 3D DL features from the tumor regions based on CT images. We utilized an MLP classifier to construct traditional radiomic models, 2D DL models, 3D DL models, and a combined model to predict the status of LVI. The results indicated that the combined model exhibited robust predictive performance. In the training, validation, and external testing sets, the combined model demonstrated the best predictive efficacy for preoperative LVI status in invasive LUAD, with AUC values of 0.958 (95%CI:0.9294 - 0.9863), 0.886 (95%CI: 0.7938 - 0.9786), and 0.884 (95%CI: 0.8277 - 0.9401), respectively. This study is the first to extract multiple radiomic models for predicting LVI status. Through a comparison of model performance, we identified the optimal model, providing quantitative support for clinical decision-making regarding surgical approaches and selecting patients requiring chemotherapy postoperatively.

Currently, radiomics plays a significant role in the preoperative assessment of lung cancer. Many researchers have applied 2D and 3D traditional radiomics to predict the status of LVI in lung cancer. The 2D radiomics has achieved remarkable results. In the studies by Yang, Nie et al. (11, 12), after modeling, the AUC of the training group reached 0.938, and that of the test group was 0.856. Theoretically, 3D radiomics can present the tumor in three dimensions and has significant advantages. However, in clinical practice, it encounters problems such as complex model construction, high computational power requirements, and deviations in the actual effect. Its practical effectiveness still needs to be explored. These studies provide multi-dimensional references for the application of radiomics in tumor imaging.

This study is the first to utilize DL to predict LVI in T1-stage invasive LUAD. Unlike previous studies, we compared traditional radiomics’ performance with DL in predicting LVI in LUAD and assessed the performance of 2D and 3D DL in this context. Theoretically, DL can directly extract raw features from tumors, potentially offering better efficacy than Rad, and 3D features may provide greater reproducibility than 2D features. However, we obtained a fundamentally unexpected result. After undergoing t-tests, Pearson correlation analysis, and LASSO, traditional radiomics, 2D DL, and 3D DL feature sets were ultimately filtered to yield 36, 31, and 6 optimal features, respectively. Only 6 3D deep learning features were retained after LASSO selection, which may be attributed to the high information concentration of 3D features—each 3D feature can integrate comprehensive information from multiple low-dimensional features in traditional radiomics. It could also be because 3D features learned based on three-dimensional spatial correlations have significantly lower information overlap between features compared to traditional radiomic features or 2D deep learning features. In the internal validation set, the Rad model outperformed the DL models, with the 2D model being more effective than the 3D model.

In this study, the traditional radiomics model outperforms DL models, with three main reasons: In terms of feature interpretability, the 44 texture features selected for radiomics(including GLCM, GLSZM, and LBP) are extracted via well-defined algorithms and have clear physical meanings (for example, GLCM can reflect the complexity of internal tumor textures, while LBP is relatively sensitive to marginal spiculation). These features provide an intuitive basis for model decision-making. In contrast, the features automatically learned by DL models are highly abstract; even with visualization techniques, their meanings are difficult to clarify, thus affecting clinical acceptance. Regarding differences in regional analysis, the 2D DL model only focuses on the maximum cross-section of the tumor, ignoring information from other layers. Meanwhile, the tumor-containing cube used in the 3D DL model often includes normal lung tissue, which interferes with feature learning. In comparison, radiomics extracts features from the entire tumor region, integrates multi-layer information, and precisely targets the tumor itself, thereby reducing interference from normal tissues. Regarding data adaptability, DL models have strict data volume and consistency requirements. Differences in CT equipment parameters among multi-center data in this study led to a significant decline in their performance on the external test set. On the contrary, radiomics mitigates the impact of equipment differences through standardized preprocessing. Additionally, its features are designed based on statistical rules, which grant stronger tolerance to data variations and enable more stable cross-center generalization. Furthermore, the 2D and 3D DL models exhibit comparable performance, which is consistent with the findings of Ma et al. (22) in head and neck tumor segmentation.

The multivariate analysis revealed that STAS (spread through air spaces) and pathological grading are independent key predictors of LVI (STAS: OR = 2.751, 95%CI=1.223-6.190, P = 0.040; pathological grading: OR = 0.403, 95%CI=0.237-0.685, P = 0.005). This finding is consistent with previous studies demonstrating close associations of STAS and pathological differentiation with tumor invasiveness and vascular invasion potential (23–26), further validating the rationality and utility of our model in integrating clinicopathological information. Specifically, as a unique airspace dissemination pattern in lung adenocarcinoma, STAS and LVI, though distinct invasive pathways, often coexist in highly aggressive tumors, indicating that tumor cells possess both trans-alveolar dissemination and vascular invasion capabilities (23, 24). Poorly differentiated tumors are more prone to vascular invasion due to active proliferation, reduced expression of adhesion molecules, and related genetic mutations (27, 28), which also explains the significant association between pathological grading and LVI.

In this study, the combined model integrating Rad features with 2D and 3D DL features can effectively predict the preoperative LVI status in patients with T1-stage invasive LUAD, providing critical references for clinical decision-making and demonstrating high application value. Although differences in CT scanning parameters across centers may lead to variations in image features, future efforts will enhance model adaptability through cross-device data augmentation. The popularity of high-performance GPUs and the Onekey AI software has simplified operations. Currently, addressing the timeliness of clinical diagnosis is essential; it is necessary to integrate the model into existing clinical imaging systems, develop a one-click analysis function, and provide operational training for radiologists. This ensures that the total time from CT image input to result output is controlled within 10 minutes, meeting the requirements of clinical timeliness. With technological advancements, this combined model holds broad prospects for clinical application.

### Limitations of this study

This study is a retrospective analysis. Due to its reliance on previous clinical data, it is prone to selection bias and grouping bias in patient screening and grouping, caused by enrollment deviations and data differences, which affect the validity and extrapolability of the conclusions. In subsequent research, biases can be reduced through prospective design, strict inclusion and exclusion criteria, and supplementation of multi-center data. The lack of follow-up data makes it impossible to evaluate the correlation between LVI prediction and patients’ actual outcomes, which limits the clinical impact of the model. Further adoption of a prospective study design combined with survival analysis will greatly enhance the translational relevance of the research. In addition, manual segmentation was used in this study. Although the segmentation procedures were carefully detailed and multiple radiologists were involved to enhance robustness, inter-reader variability that may still exist due to the reliance on manual segmentation could influence the model’s generalizability. In future iterations of the research, models will be trained on automatically segmented regions. This study focused solely on the tumor region, neglecting the peritumoral area; further research will address the peritumoral region. In summary, we will refine the study protocol to enhance the stability of the model and broaden its applicability.

## Conclusion

This study combined Rad and DL models to predict the LVI status in patients with T1-stage invasive LUAD. The combined model demonstrated significant potential as a clinical tool due to its robust predictive capability. It provides a more accurate prediction of LVI status in T1-stage invasive LUAD, offering more substantial evidence to guide surgical decision-making and the need for postoperative chemotherapy. The model’s robust performance has been validated through stable AUC results in the external testing cohort.

## Data Availability

The original contributions presented in the study are included in the article/supplementary material. Further inquiries can be directed to the corresponding authors.
